# Neutrophlic dermatosis (Sweet's syndrome-like) induced by paracetamol

**DOI:** 10.1186/2045-7022-4-S3-P83

**Published:** 2014-07-18

**Authors:** M Teresa Dordal Culla, Ramon Navarra Amayuelas, M Teresa Martin-Urda Diez-Canseco, M Teresa Fernandez-Figueras, Carles Lucas Giralt, M Mercedes Gomez Vazquez

**Affiliations:** 1 Hospital Municipal de Badalona, Allergy Department, Spain; 2 Hospital Municipal de Badalona, Dermatology Department, Spain; 3 Hospital Germans Trias i Pujol, Pathology Department, Spain

## Background

Paracetamol (PC) is one of the most popular analgesics and antipyretics. It is generally considered a safe drug, although it can occasionally induce hypersensitivity reactions.

## Case-report

An 82 year-old man with hypertension and ischemic heart disease underwent an inguinal hernioplasty. Twenty hours after initiating PC administration in the surgery room, a slightly pruritic eruption suddenly appeared with multiple tender, oedematous and erythematous plaques on the trunk and extremities, associated to general malaise, headache and myalgia. He didn't seek for medical care until 1 week later, when he came to the hospital for suture removal. No treatment was prescribed and the eruption resolved in 4 weeks. Two months later the patient developed, once again, a similar generalized skin eruption with fever (38.5ºC), general malaise and weakness. Neutrophilia (88.6%) and elevated C-reactive protein levels were detected. There was no mucosal or extracutaneous involvement. Few hours previously he had taken PC for a mild non-pyretic upper respiratory tract infection. The drug was withdrawn, and all signs and symptoms resolved after a short course of corticosteroid treatment. No evidence of active infection was detected. Histological examination of a skin biopsy specimen showed a neutrophilic infiltrate with mild eosinophilia, neutrophilic spongiosis and lichenoid damage. Five months later we performed epicutaneous patch test with PC in unaffected and previously affected skin, with negative results. The patient refused to perform more allergic studies. He had been tolerating ibuprofen, metamizole and acetylsalicylic acid after the last reaction. Neither new episodes nor evidence of malignancy have been detected in the meantime.

## Observations

The abrupt onset and type of cutaneous lesions, associated fever and neutrophilia, histopathological results, prompt response to corticosteroid therapy and drug withdrawal, and previous history of a similar reaction with PC intake, suggest a neutrophilic dermatosis (Sweet's syndrome-like) induced by PC.

